# Epstein-Barr Virus Nuclear Antigen 1 Recruits Cyclophilin A to Facilitate the Replication of Viral DNA Genome

**DOI:** 10.3389/fmicb.2019.02879

**Published:** 2019-12-13

**Authors:** Shuyu Xin, Shujuan Du, Lingzhi Liu, Yan Xie, Lielian Zuo, Jing Yang, Jingjin Hu, Wenxing Yue, Jing Zhang, Pengfei Cao, Fanxiu Zhu, Jianhong Lu

**Affiliations:** ^1^NHC Key Laboratory of Carcinogenesis, The Key Laboratory of Carcinogenesis and Cancer Invasion of the Chinese Ministry of Education, Department of Hematology, Xiangya Hospital, Central South University, Changsha, China; ^2^Department of Medical Microbiology, School of Basic Medical Science, Central South University, Changsha, China; ^3^Department of Biological Sciences, Florida State University, Tallahassee, FL, United States

**Keywords:** Epstein-Barr virus nuclear antigen 1, latent genome, cyclophilin A, replication, persistence, pathogenesis

## Abstract

Epstein-Barr virus (EBV) nuclear antigen 1 (EBNA1)-mediated DNA episomal genome replication and persistence are essential for the viral pathogenesis. Cyclophilin A (CYPA) is upregulated in EBV-associated nasopharyngeal carcinoma (NPC) with unknown roles. In the present approach, cytosolic CYPA was found to be bound with EBNA1 into the nucleus. The amino acid 376-459 of the EBNA1 domain was important for the binding. CYPA depletion attenuated and ectopic CYPA expression improved EBNA1 expression in EBV-positive cells. The loss of viral copy number was also accelerated by CYPA consumption in daughter cells during culture passages. Mechanistically, CYPA mediated the connection of EBNA1 with oriP (origin of EBV DNA replication) and subsequent oriP transcription, which is a key step for the initiation of EBV genome replication. Moreover, CYPA overexpression markedly antagonized the connection of EBNA1 to Ubiquitin-specific protease 7 (USP7), which is a strong host barrier with a role of inhibiting EBV genome replication. The PPIase activity of CYPA was required for the promotion of oriP transcription and antagonism with USP7. The results revealed a strategy that EBV recruited a host factor to counteract the host defense, thus facilitating its own latent genome replication. This study provides a new insight into EBV pathogenesis and potential virus-targeted therapeutics in EBV-associated NPC, in which CYPA is upregulated at all stages.

## Introduction

Epstein-Barr virus (EBV) is a member of the gamma herpesviruses and was the first confirmed human tumor virus (Ding et al., 2013; Lu et al., 2017). EBV ubiquitously infects more than 90% of the global population and is closely associated with the development of several malignancies, including Burkitt’s lymphoma and nasopharyngeal carcinoma (NPC) (Shair et al., 2008; Yu et al., 2012; Young, 2014; Young and Dawson, 2014; Liu et al., 2017). NPC, which is primarily of epithelial origin, is a type of metastatic head-and-neck neoplasm that is highly prevalent in southern China and some other areas in East Asia and Africa (Tang et al., 2016; Tu et al., 2017; Jiang et al., 2018). EBV is able to establish life-long persistence in the human host (Young and Murray, 2003; Zuo L. et al., 2017). It contains a large genome approximately 172 kb in size and has two phases in its life cycle (the latent and lytic stages) (Yu et al., 2011; Hammerschmidt and Sugden, 2013). EBV mainly spreads through the saliva of human host, infects B cells through the oral mucosal epithelium, and then transforms B lymphocytes into resting memory B cells through a series of viral latent transcription programs, thus establishing a lifelong latent infection pattern (Thorley-Lawson et al., 2013). The majority of EBV infections *in vivo* are latent (Thorley-Lawson, 2015). During EBV latency, the EBV genome exists in the form of episome DNA, few viral genes are expressed, and no virion is produced. EBV nuclear antigen 1 (EBNA1) is the only viral protein that is expressed in all types of EBV-associated tumors (Lu et al., 2010; Tao et al., 2015). Determining how EBV is able to maintain its stable latent status in host cells is a topic of interest, because it may provide understanding about the pathogenesis of EBV and new targets to inhibit the persistence of EBV genome in the therapy of EBV-associated cancers. EBV replication is under the control of some host and viral factors that are not fully understood. EBNA1 plays a key role in the replication and mitotic segregation of EBV DNA episomes to daughter cells (Yates and Guan, 1991; Frappier, 2012b). EBNA1-mediated S-phase episome replication depends on binding of EBNA1 to the EBV origin of genome replication (oriP) (Reisman et al., 1985). Viruses are obligate intracellular parasites, and their replication cycles depend on some host cell factors. For example, some studies suggested that cellular origin recognition complex (ORC) and minichromosome maintenance (MCM) complex are related to the DS element of oriP, implicating them in the initiation of EBV DNA replication (Frappier, 2012a; Capone et al., 2015). These host factors may also be potential targets for antiviral therapy.

Cyclophilin A (CYPA) is a protein with multiple functions as a typical member of the cellular peptidyl-prolyl *cis*-*trans* isomerase (PPIase) family (Braaten et al., 1996; Bahmed et al., 2012). CYPA was discovered initially as an intracellular receptor of the immunosuppressive drug cyclosporin A (CsA) (Braaten et al., 1996; Bahmed et al., 2012). Studies have shown that CYPA can use IL-6 to induce cell signal conversion, activate tyrosine phosphorylation and nuclear transport of transcription factor 3, and can bind and activate NF-κB (Tang et al., 2015). CYPA is involved in the life cycles of multiple viruses and plays a critical role in their successful infectivity and replication, including human immunodeficiency virus type 1 (HIV-1), hepatitis C virus (HCV), hepatitis B virus (HBV), vesicular stomatitis virus (VSV), vaccinia virus (VV), coronaviruses (CoVs), and feline coronavirus (Bose et al., 2003; Naoumov, 2014; Jyothi et al., 2015; Phillips et al., 2015). The interaction between CYPA and HIV protein promotes the replication and infection of HIV particles; CD147 is the main signal receptor of CYPA, and the two interact to regulate the early steps of HIV replication (Ciesek et al., 2009; Tang et al., 2015). Conversely, CYPA suppresses the replication of some viruses, such as rotavirus, infectious bursal disease virus and influenza virus (Xu et al., 2010; Liu et al., 2012). However, the role and mechanism of CYPA in the function of EBV remain unknown. Our laboratory previously performed a proteomics study using NPC tissues and found that CYPA was upregulated from the early stages of NPC (atypical hyperplasia and stage I) to the malignancy stages (Yang et al., 2014). Since EBV infection also occurs during the early stage of NPC (Morrison et al., 2004), we speculated that a potential relationship might exist between CYPA and EBV, which initiated the present approach. Recently, we have reported that the exosomal CYPA level in NPC is positively related that of exosomal latent membrane protein 1, which is another latent protein of EBV. The result suggests a relationship between CYPA and EBV (Liu et al., 2019).

Ubiquitin-specific protease 7 (USP7) is a type of deubiquitinase that is also known as herpesvirus-associated ubiquitin specific protease (HAUSP) and has been documented as a host factor that inhibits EBV replication (Holowaty et al., 2003a). The USP7-EBNA1 interaction was selectively disrupted by deletion of EBNA1 residues 395–450 just upstream of the DNA binding domain, and the resulting EBNA1 mutant exhibited significantly increased DNA replication activity (Holowaty et al., 2003b). USP7 also plays a role in the replication inhibition of Kaposi’s sarcoma-associated herpesvirus (KSHV) by interacting with the latency-associated nuclear antigen 1 (LANA), which is the homology of EBNA1 (Jager et al., 2012). As it is known, KSHV also persists in infected cells mainly in a latent state, and LANA, is expressed in all latently KSHV-infected cells (Rainbow et al., 1997). However, the mechanism by which EBV overcomes host suppression to maintain its own pathogenesis remains to be investigated.

Here, we demonstrate that EBNA1 binds to and recruits CYPA to the nucleus to support the function of EBNA1 in the replication of the viral latent genome. CYPA overexpression could antagonize the host barrier, USP7. The results revealed a strategy that EBV recruited one host factor to counteract another, thus favoring the viral DNA replication and persistence in latent infection. The study provides novel insights into understanding EBV pathogenesis in epithelial cells.

## Materials and Methods

### Cell Lines and Plasmids

The p2089 plasmid (Maxi-EBV), which contains the complete EBV genome of the B95–8 strain, was kindly provided by Dr. W Hammerschmidt (Delecluse et al., 1998). The human embryonic kidney HEK293 cell line (HEK293) was originally obtained from ATCC and was used to establish latent infection of the whole EBV genome (p2089) by using hygromycin for the screening, resulting in the C2089 cell line that was previously described by our group (Zuo et al., 2015). Subsequently, the cells were divided into groups of shRNA (using pRNAT-U6.1-shRNA-CYPA treated cells), the NC shRNA group (pRNAT-U6.1-shRNA treated cells CYPA NC), Liposomal transfected cells are based on Lipofectamine 3000 transfection reagent (Invitrogen-Thermo Fisher; United States). The day after transfection, cells were treated with G418 was added (Sigma-Merck; Germany) for cell selection. Cells were subcultured every 2 days. The cells were grown in Dulbecco’s modified Eagle’s medium (DMEM; Sigma) supplemented with 10% FCS. The C666-1 cell line is an EBV-positive NPC cell line (Zuo L.L. et al., 2017).

All recombinant plasmids used for bimolecular fluorescence complementation (BiMC) were constructed using standard cloning techniques according to the schematic diagram (Supplementary Figure S1) by using the pCAGGS vector, which is a gift from Dr. Harty (Lu et al., 2013). CYPA and CYPB were amplified from 93 cells by RT-PCR. CYPA or CYPB gene was fused with the N-terminal fragment of the yellow fluorescent protein (YFP), constructing the plasmids of pCAGGS-Flag-CYPA-NY (CYPA-NY) and pCAGGS-Flag-CYPB-NY (CYPB-NY), respectively. The EBNA1 protein was expressed as a fusion protein with the C-terminal fragment of (YFP), constructing the plasmid pCAGGS-Myc-EBNA1-CY (EBNA1-CY). The expression plasmids pCAGGS-Flag-CYPA and pCAGGS-Flag-CYPB were also constructed respectively. The Myc tag sequence was added to the N-terminal of EBNA1. Myc-tagged EBNA1 was generated by cloning into the *Sph*I and *Nhe*I sites of the pCAGGS vector to produce the pCAGGS-Myc-EBNA1 plasmid. The truncations EBNA1 (NT, 1-90) and EBNA1 (607-641) were generated by PCR based on the plasmid pCAGGS-Myc-EBNA1 using the primers listed in Supplementary Table S1. The EBNA1 deletion mutants EBNA1 (NT, 90-376), EBNA1 (376-459) and EBNA1 (459-607) were generated by inverse PCR. For example, primer complementary to 243–67 bp (anti-sense) and 1129–1156 bp (sense) of EBNA1 were used for amplification, and the resulting PCR.

product was gel-purified and self-ligated, resulting in EBNA1 (Δaa 90-376). Then a *Sph*I-*Nhe*I fragment from EBNA1 (Δaa 90-376) was inserted into the *Sph*I and *Nhe*I sites of the pCAGGS-Myc-EBNA1 to produce the pCAGGS-Myc-EBNA1 (Δaa 90-376).

The oriP-SV40-Luc expression vector was obtained by introducing the flanking sequence of oriP amplified from the pc3-oriP plasmid, which was a gift from Prof. Frappier (Sivachandran et al., 2011). The oriP sequence was cloned into the *Sac*I and *Xho*I sites of the luciferase reporter vector, PGL3-enhancer.

### RT and Real-Time Quantitative PCR (RT-qPCR)

The RT-qPCR was performed as previously described (Yu et al., 2012). Briefly, total RNA was isolated from cells using the TRIzol reagent (Invitrogen). First, TRIzol was added to lyse the cells, then 1/5 volume of chloroform was added to the lysate to separate the DNA, protein and RNA, followed by the isopropanol and 75% absolute ethanol to precipitate the RNA. For RT, 1 μg of RNA was reversely transcribed into cDNA using the One-Step gDNA Removal and cDNA Synthesis SuperMix kit (TransGen Biotech, Beijing, China). Real-time quantitative PCR (RT-qPCR) was then performed by using the kit of *TransStart*^®^ Top Green qPCR SuperMix (TransGen Biotech, Beijing, China) according to the manufacturer’s instructions. The following program was used for the RT reaction: 65°C for 5 min, ice for 2 min, followed by 42°C for 15 min and then 85°C for 5 s. The CFX Multicolor Detection System (Bio-Rad) was employed for the detection. Primers for RNA detection by qRT-PCR were designed based on RNA sequences, and bate-actin was used as an endogenous control. The following procedure was used for the RT-qPCR: 95°C for 3 min, followed by 40 cycles of 95°C for 12 s and 62°C for 35 s. Data obtained by the conventional method of calculating comparative method 2^(–ΔΔct)^. Repeated three times for each sample in parallel in each experiment, and the results were expressed as the mean of three independent experiments. The sequences of the qRT-PCR primers are provided in Supplementary Table S1.

### Western Blotting Analysis

Western blotting (WB) was performed using a standard protocol as described previously (Zuo L.L. et al., 2017). cells was lysed in RIPA (50 mM Tris, pH 7.4, 150 mM NaCl, 1% NP-40, 0.5% sodium deoxycholate, 0.1% SDS, sodium orthovanadate, sodium fluoride, EDTA, leupeptin) followed by incubation on ice for 30 min, protein quantification according to the specification of BCA kit (Beyotime, Shanghai, China). The cells lysates were centrifuged at 12000 × *g* for 20 min at 4°C. Supernatant fractions were used for detection. Samples with equivalent amounts of denatured protein were separated using 10% SDS polyacrylamide gels (Epizyme, Shanghai, China). After electrophoretic separation, the proteins were transferred to a polyvinylidene difluoride (PVDF) membrane (Millipore, Danvers, MA, United States). The membrane was blocked with 5% skimmed milk for 1 h at room temperature, followed by an overnight incubation with the primary antibody at 4°C. After washing three times, the membrane was incubated with a secondary antibody for 1 h at 37°C. Finally, the proteins of interest were detected using the Luminata Crescendo HRP Substrate (Millipore) and viewed with the ChemiDoc XRS + Molecular Imager (Bio-Rad). The results were analyzed using the Image Lab software (Bio-Rad).

The following antibodies were used for the immune detection: anti-EBNA1 (Santa Cruz Biotech, DE, United States, sc-81579), anti-CYPA (Proteintech, Chicago, IL, United States, 10720-1-AP), anti-C-Myc (Sigma, St. Louis, MO, United States, C3956), and anti-Flag (Sigma, H9658). GAPDH (Proteintech, Chicago, IL, United States, 10494-I-AP) and bate-actin (Proteintech, Chicago, IL, United States, 66009-I-Ig) were used as the loading controls. The secondary antibodies used for WB and IF were as follows: HRP-conjugated anti-rabbit (CST, Chicago, IL, United States, #7074P2), HRP-conjugated anti-mouse (GE Healthcare, United Kingdom, NA931V) and Alexa Fluor 488 donkey anti–rabbit IgG (Life technologies, United States, 1480770).

### Cell Counting Kit-8 Assay

C2089 cells were plated in a 96-well plate at a density of 1 × 10^3^ cells per well. The next day, different CsA concentrations as indicated were applied for treatment. At 24, 48 and 72 h post-treatment, the Cell Counting Kit-8 (CCK8) was used to test cell viability according to the manufacturer’s instructions. The absorbance was detected at the 450 nm wavelength using a microplate reader.

### Cyclosporin A (CsA) Treatment

Cyclosporin A (Dalian Meilun, China) is a type of immunosuppressive preparation that targets CYPA (Chatterji et al., 2009). The drug was dissolved in dimethyl sulfoxide (DMSO). EBV-positive cells were cultured in medium containing 40 μM of CsA. After 48 h of treatment, the cellular proteins and total RNA were extracted and subjected to detection.

### Bimolecular Fluorescence Complementation (BiMC) Assay

Bimolecular fluorescence complementation assay was performed as described previously (Liu et al., 2011; Lu et al., 2013, 2014). The schematic diagram of BiMC assay is shown in Supplementary Figure S1. In brief, human HEK293T cells were grown on coverslips in six-well plates. The pCAGGS-Myc-EBNA1-CY plasmid or the mutant constructs and the pCAGGS-Flag-CYPB-NY plasmid were cotransfected into the cells using the Lipofectamine 3000 transfection reagent (Invitrogen) according to the manufacturer’s instructions. Approximately 0.25 μg of each plasmid was used for the transfection. Transfection of a single plasmid for each used in BiMC assay was performed as a negative control. After 24 h, the cells were gently washed for three times with PBS and fixed with cold 4% paraformaldehyde for 30 min at room temperature. Then, the cells were washed and subsequently stained with Hoechst 33342 (Sigma) for 3 min at room temperature. Finally, the cells were washed again, and the slides were observed for the specific YFP signal under a fluorescence microscope.

### Co-immunoprecipitation (co-IP)

A typical co-IP procedure was performed as described (Liu et al., 2011; Lu et al., 2013, 2014). Cell lysates were harvested directly or at 48 h post-transfection as indicated. The lysed sample was centrifuged at 13,000 rpm for 20 min at 4°C. The supernatant lysates of about 1 mg were incubated with a primary antibody, such as an anti-Flag monoclonal antibody (mAb) (H9658, Sigma, 1:100) or anti-mouse IgG antibody (control) (sc-0025, Santa Cruz, 1:200), for 10 h at 4°C. After centrifugation, the supernatant was transferred to a new tube containing 40 μl of protein G beads (TransGen Biotech, Beijing, China) and incubated for 6 h at 4°C. After extensive washes with cold lysis buffer, the immunoprecipitated proteins were eluted in SDS sample loading buffer (Aurigene Biotech, Changsha, China), separated by SDS-PAGE, transferred onto polyvinylidene difluoride membranes (Millipore), and detected by WB (Zheng et al., 2012).

### RNA Interference

Three small interfering RNAs (siRNAs) targeting CYPA (GenBank accession number: NM 001166326) were designed and synthesized by Guangzhou RIBOBIO Company. To evaluate the knock-down efficiency of the siRNAs, 100 pmol of each siRNA was transfected into HEK293 cells. The cells were lysed with RIPA as described by WB, and lysates were used for western blotting with anti-CYPA and anti-bate-actin antibodies. The siRNA with the best knock-down efficiency was chosen for the subsequent experiments. A scramble siRNA-NC was used as the control.

### Immunofluorescence (IF) Assay

HEK293T and Vero cells grown on coverslips in six-well plates for 24 h were washed gently three times with PBS, fixed with 4% formaldehyde in PBS for 15 min, and then permeabilized with 0.5% Triton X-100 in PBS for 5 min. Subsequently, the cells were blocked with fresh 10% goat serum. The cells were incubated with an anti-CYPA rabbit polyclonal antibody and anti-Flag mouse mAb overnight at 4°C, respectively. After washing five times with PBS, the secondary antibody was added for 1 h of incubation at 37°C. Then, Hoechst 33342 was applied for nuclear staining for 3 min at room temperature. The coverslips were finally mounted onto slides and observed under a florescent microscope (BX53, Olympus, Japan).

### Chromatin Immunoprecipitation (ChIP) Assay

The ChIP assay was performed to detect the binding of EBNA1-oriP according to previous reports (Shen et al., 2016). The Immunoprecipitation Kit (Millipore) was used for the assay. HEK293 cells were transfected with Lipofectamine 3000 (Invitrogen) according to the manufacturer’s protocol. Cells were collected after 48 h and lysed with lysis buffer (Beyotime, Shanghai, China) [20 mM Tris (pH7.5), 150 mM NaCl, 1% Triton X-100, sodium pyrophosphate, β-glycerophosphate, EDTA, Na_3_VO_4_, leupeptin]. The immunoprecipitated nucleoprotein complexes were eluted by incubation twice for 15 min at 25°C with 200 μl of elution buffer (1% SDS and 100 mM NaHCO_3_), and the crosslinks were reversed by incubation at 65°C for 4 h. The DNA was extracted with phenol/chloroform and precipitated with ethanol. Then oriP DNA was amplified by qRT-PCR using the primers listed in Supplementary Table S1.

### Luciferase Reporter Assay

The experimental procedure for the detection of oriP transcription activation mediated by EBNA1 was carried out according to previous description from other group (Chen et al., 2014). Cells were seeded into 24-well plates 24 h prior to transfection. The following day, 1000 ng of the orip-SV40-Luc reporter plasmid was transfected into the cells using Lipofectamine 3000. The SV40-Luc reporter plasmid was transfected as a control. Cell lysates were collected at 24 h post-transfection and assayed for luciferase activity using a luciferase assay kit (Promega, Madison, WI, United States) on the Panomics Luminometer. The Renilla luciferase activity was also measured using an enzyme assay kit (Promega). The results were normalized to the Renilla activity. Three parallel repeats were performed for each sample in each experiment, and the results were expressed as the mean of three independent experiments.

### Detection of the EBV Copy Number

The detection of EBV copy number was performed as described previously (Zuo et al., 2015). DNA was extracted from the C2089-shNC and C2089-shCYPA cells using the General AllGen Kit (CWBio, Hunan, China) and quantified. The relative EBV copy number was determined by RT-qPCR using the EBV DNA Quantitative Fluorescence Diagnostic Kit (Sansure Biotech, Hunan, China) according to the manufacturer’s instruction. In this product, *Bam*HI-W fragment in EBV genome is designed as the specific primers and probes. The EBV copy number concentration (copies/cell) of the samples was calculated according to the level of internal reference. The experiment was repeated for three times.

### Statistical Analysis

The statistical analyses were performed using GraphPad Prism 5 (GraphPad Software, CA, United States). Differences between groups were determined using Student’s *t*-test or one-way analysis of variance (ANOVA). The data are expressed as the means ± standard deviations (SDs). Single, double and triple asterisks indicate statistical significance (^∗^*P* < 0.05, ^∗∗^*P* < 0.01 and ^∗∗∗^*P* < 0.001).

## Results

### CYPA, but Not CYPB, Binds to EBNA1

**FIGURE 1:**
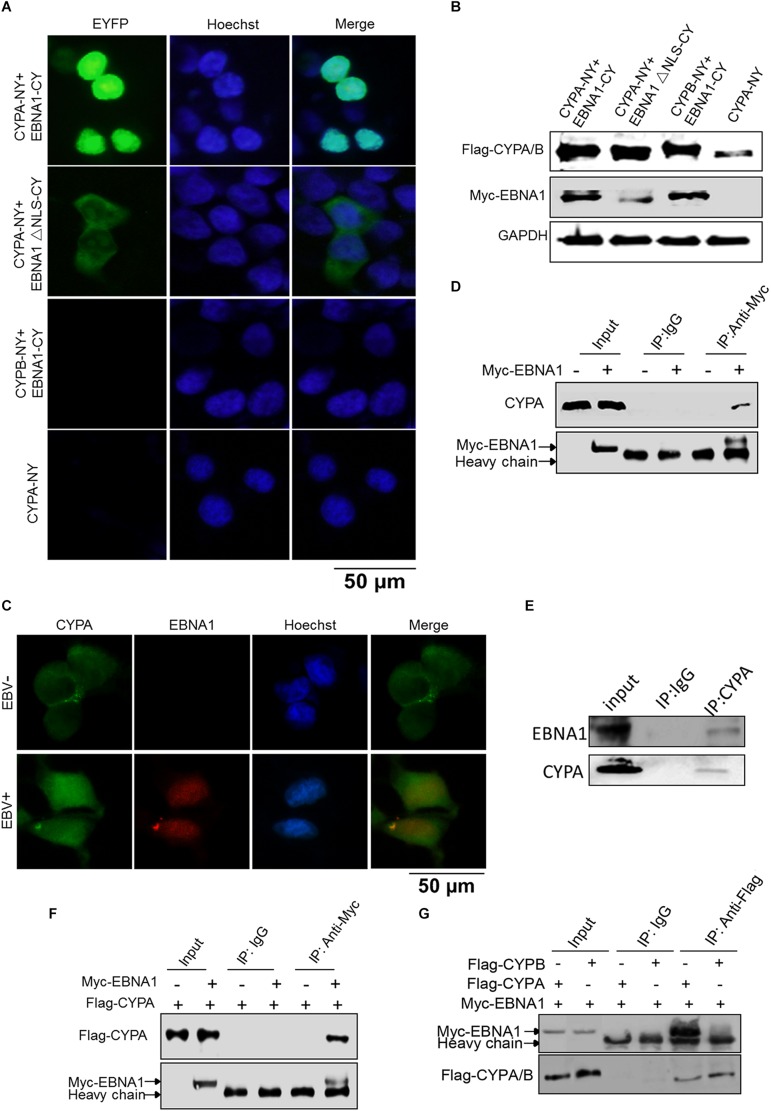
Detection of CYPA-EBNA1 binding by the BiMC and co-IP assays. **(A)** Detection of the EBNA1-CYPA interaction by the BiMC assay. HEK293T cells were transfected with the indicated plasmids, including CYPA-NY, EBNA1-CY, EBNA1ΔNLS-CY, and CYPB-NY following by BiMC analysis, and fluorescence was observed. Transfection of a single CYPA-NY plasmid did not lead to the production of fluorescence. Scale bar, 50 μm. **(B)** The proteins expressed from the plasmids used in the BiMC assay were detected by WB. **(C)** The detection of CYPA and EBNA1 in EBV-negative and EBV-positive HEK293 cells by IF assay. Scale bar, 50 μm. **(D)** Endogenous CYPA interacts with EBNA1 in HEK293 cells. The plasmid pCAGGS-Myc-EBNA1 was transfected into cells. An anti-Myc antibody was used for the pull-down the CYPA, and the WB assay was carried out for detection. **(E)** Endogenous CYPA interacts with EBNA1 in C2089 cells. EBNA1 was immune-precipitated with anti-CYPA. EBNA1 was detected by WB **(F)** Exogenous CYPA interacts with EBNA1. HEK293 cells were transiently transfected with Flag-CYPA alone or with Myc-EBNA1. Flag-CYPA was immune-precipitated with anti-Myc antibody. IgG was used as a negative control for the pull-down in the co-IP assay. Flag-CYPA was detected by WB. **(G)** Co-IP assay for comparison of the interactions of CYPA and CYPB with EBNA1. PCAGGS-Myc-EBNA1 and pCAGGS-Flag-CYPA or pCAGGS-Flag-CYPB were transfected in HEK293 cells. Myc-EBNA1 was immune-precipitated with anti-Flag antibody.

By immunofluorescence (IF) assay, CYPA was detected to be mainly localized in the cytoplasm in Vero and HEK293T cells (Supplementary Figure S2). To study the potential interaction between EBNA1 and CYPA, BiMC assay was performed. The BiMC assay is a useful tool for detection of protein-protein interactions in living cells, and the results can be visualized under a fluorescence microscope (Hudry et al., 2012; Yang et al., 2014). A diagram of the basic principle of the BiMC assay is shown in Supplementary Figure S1. The EBNA1 protein was expressed as a fusion protein with the C-terminal fragment of the YFP (pCAGGS-Myc-EBNA1-CY), and CYPA was fused with the N-terminal fragment of YFP (pCAGGS-Flag-CYPA-NY). These two plasmids were co-transfected into HEK293T cells, with the single pCAGGS-Flag-CYPA-NY plasmid transfection as a negative control. The results showed that EBNA1 and CYPA interacted with each other in the nucleus (Figure 1A, Top). When the nuclear localization signal (NLS) sequence of EBNA1 was deleted, the proteins interacted in the cytoplasm (Figure 1A). This translocation showed the specificity of the interaction mediated by EBNA1 with an NLS. Cyclophilin B (CYPB) is another member of the cyclophilin family, but we did not detect any binding signal for CYPB-EBNA1 in the BiMC assay (Figure 1A). There was no fluorescence signal in the cells transfected with only single plasmid which was a negative control (Figure 1A, Lower). EBNA1 and CYPA/B expression in the BiMC assay was detected by WB (Figure 1B). We further evaluated EBNA1 and CYPA intracellular localization in EBV-positive cells by IF assays (Figure 1C). The result showed that CYPA expressed in both cytoplasm and nucleus in EBV-positive C2089 cells, while mainly in the cytoplasm of the EBV-negative HEK293 cells (Figure 1C). The co-immunoprecipitation (co-IP) assay was used to verify the interaction of EBNA1 with CYPA. These results showed that both endogenous and exogenous CYPA interacted with EBNA1 (Figures 1D–F). The plasmids pCAGGS-Myc-EBNA1 and pCAGGS-Flag-CYPA or pCAGGS-Flag-CYPB were transfected in HEK293 cells, Myc-EBNA1 was immune-precipitated with anti-Flag antibody. As shown in Figure 1G, the result of co-IP assay further validated that EBNA1 interacted with CYPA but not CYPB.

### EBNA1 Domain With Amino Acids 376-459 Is Essential for the CYPA-EBNA1 Interaction

**FIGURE 2:**
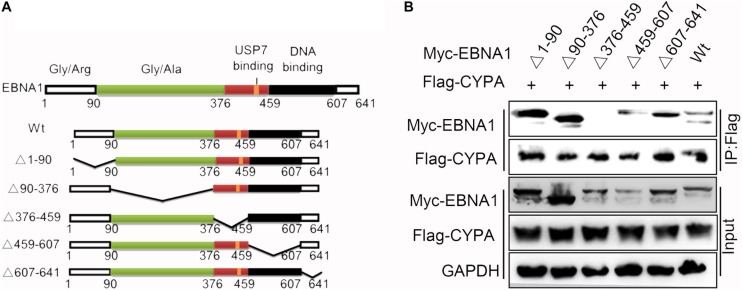
Identification of the EBNA1 domain required for binding to CYPA. **(A)** Diagram of the EBNA1 deletion mutants. The start and end amino acid residues for each fragment are indicated according to a previous report (Young and Murray, 2003). **(B)** Validation of the interaction between each mutant EBNA1 and CYPA by the co-IP assay. PCAGGS-Myc-EBNA1, pCAGGS-Myc-EBNA1 mutants, and pCAGGS-Flag-CYPA were transfected into HEK293 cells. Myc-EBNA1 was immune-precipitated with anti-FLAG antibody. Flag-CYPA and Myc-EBNA1 were detected by WB.

To identify the contribution of the functional domains of EBNA1 to the CYPA-EBNA1 interaction, we constructed five deletion mutants of Myc-tagged EBNA1 named EBNA1Δ1-90, EBNA1Δ90-376, EBNA1Δ376-459, EBNA1Δ459-607, and EBNA1Δ607-641. The plasmid structures are illustrated in Figure 2A. The BiMC assay revealed that YFP was reconstructed in HEK293T cells using wild-type EBNA1 and four of the mutants (EBNA1Δ1-90-CY, EBNA1Δ90-376-CY, EBNA1Δ459-607-CY and EBNA1Δ607-641-CY) but not the EBNA1Δ376-459-CY mutant (Supplementary Figure S3A). Each negative control with single plasmid transfection is shown in Supplementary Figure S3C. Subsequently, we co-transfected pCAGGS-Myc-EBNA1 or a pCAGGS-Myc-EBNA1 mutant plasmid with pCAGGS -Flag-CYPA into HEK293 cells. A co-IP assay using a Flag-tag antibody for the pulldown showed the same result (i.e., CYPA did not interact with EBNA1Δ376-459) (Figure 2B). This domain, which is also the region containing the USP7-binding domain in EBNA1 (Figure 2A), was thus critical for the binding of EBNA1 to CYPA. Co-expression of EBNA1 376-459-CY (only this one domain of EBNA1) and CYPA-NY recovered their binding function based on the BiMC assay results (Supplementary Figure S3B). The NLS (amino acids 379-386) is also included in the domain containing amino acids 376-459, and thus YFP was detected in the nucleus (Supplementary Figure S3B). These results indicated that amino acids 376-459 of EBNA1, which contain the USP7-binding domain, were essential for the interaction between EBNA1 and CYPA.

### CYPA Influences EBNA1 Expression and Maintenance of EBV Genome During Cell Culture Passages

**FIGURE 3:**
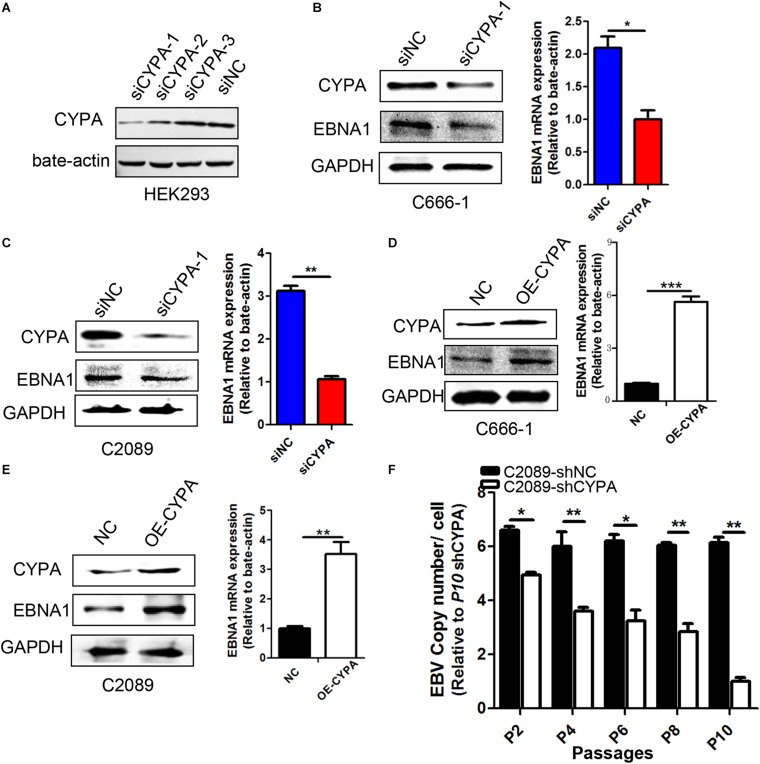
Effect of CYPA on EBNA1 expression and loss of viral copy numbers. **(A)** Designations of the CYPA siRNAs. Three siRNAs for CYPA and a control siRNA were designed and used for detection of the interference efficiency. HEK293 cells were transfected with one of the siRNAs, and a WB assay was performed at 48 h post-transfection for analysis of the CYPA protein level. **(B,C)** Effect of siCYPA on EBNA1 expression in EBV-positive cells. Two EBV-positive cell lines [C666-1 **(B)** and C2089 **(C)**] were used for siRNA transfection. At 48 h post-transfection, the cell lysates and RNA were subjected to the WB and RT-qPCR assays respectively, with siCYPA standarized to 1. **(D,E)** Effect of CYPA overexpression on EBNA1 expression at protein and mRNA levels in EBV-positive cells [C666-1 **(D)** and C2089 **(E)**]. **(F)** The effect of CYPA depletion on loss of EBV copy numbers during passages. EBV-positive C2089 was used for shRNA stable transfection and selection. Cell lines stably transfected with shRNA-CYPA or shRNA-NC were established for the experiment. Cell dispersal for each passage was performed at a ratio 1:2. The copy number of the C2089-shCYPA cells was decreased compared with the C2089-shNC, with passage 10th of C2089-shCYPA set to 1. ^∗^*P* < 0.05, ^∗∗^*P* < 0.01, ^∗∗∗^ *P* < 0.001.

We designed three siRNAs to observe their efficiency on CYPA protein expression in HEK293 cells (Figure 3A). The results showed that the knock-down of siCYPA-1 was best, and this siRNA was used in the subsequent experiments, whereas a scrambled control siRNA (siNC) had no effect on CYPA expression. As shown in Figures 3B,C, EBNA1 protein and mRNA expression decreased in response to siCYPA in EBV-positive NPC C666-1 and C2089 cells. Conversely, ectopic CYPA overexpression in the EBV-positive cell lines resulted in an increase in the EBNA1 expression levels detected by WB and qRT-PCR (Figures 3D,E).

As EBV episomal genome is easy to be lost with culture passages *in vitro* (Frappier, 2012b), we investigated whether CYPA depletion might expedite this process in consecutive passages. Then we collected gDNA from different generations of cells and detected the copy number of each cell. As it shown in Figure 3F, CYPA depletion significantly facilitated loss of EBV copy numbers.

### CYPA Regulates Transcription Initiation of the EBV Genome via Promoting the EBNA1-Mediated OriP Transcription

**FIGURE 4:**
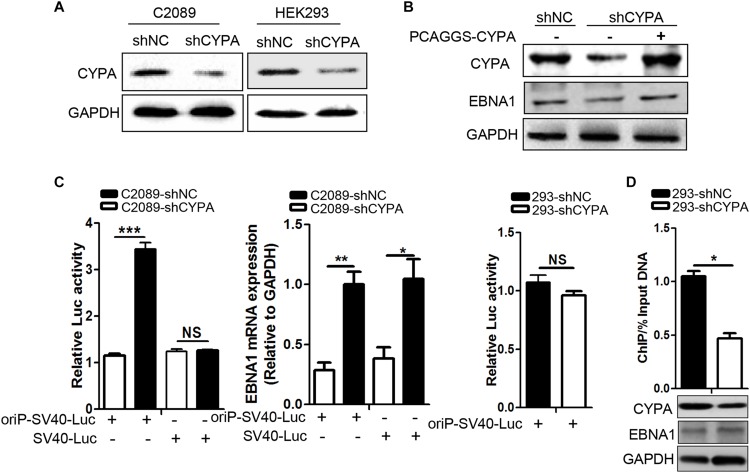
The effect of CYPA knockdown on EBNA1–mediated oriP transcription activity. **(A)** The detection of CYPA expression in stably transfected cell lines with shRNA-CYPA and shRNA-NC. **(B)** Restoration of EBNA1 protein expression in C2089-shCYPA cells transfected with wild-type CYPA expression plasmids. CYPA and EBNA1 proteins were detected by WB. **(C)** Effect of CYPA knockdown on EBNA1-oriP-mediated transcription activity in the luciferase reporter assay. The EBNA1 mRNA was detected by RT-qPCR. **(D)** Effect of CYPA knockdown on binding of EBNA1 to oriP in the ChIP assay. CYPA was depleted from HEK293 with shRNA. Antibodies against Myc and IgG control were respectively used for the pulldown in ChIP assaysHEK293. The precipitation of oriP DNA was quantitated by RT-qPCR. CYPA and EBNA1 proteins were detected by WB. ^∗^*P* < 0.05, ^∗∗^*P* < 0.01, ^∗∗∗^ *P* < 0.001.

EBNA1-oriP binding has been validated to be necessary for the replication initiation of EBV genome (Shen et al., 2016). Here, we investigated the effect of CYPA knockdown on the oriP luciferase activity in cells transfected with an oriP luciferase reporter in C2089 cells. EBV-positive C2089 and EBV-negative HEK293 cells were stably transfected with a lentivirus expressing a short hairpin (sh) of CYPA and a scramble control (shNC) (Figure 4A), and the results showed that CYPA was successfully knocked down. EBNA1 protein expression was restored in the C2089-shCYPA cells transfected with the wild-type CYPA expression plasmid (Figure 4B). In these cells, an oriP luciferase reporter, oriP-SV40-Luc was transiently transfected. After 24 h transfection, luciferase activities were detected. The EBNA1 activated oriP Luciferase was decreased by 3.5-fold in the luciferase assay compared to that in the control cells (C2089-shNC) due to CYPA depletion. In contrast, there was no effect on the transfection of an SV40-Luc reporter plasmid with CYPA depletion (*P* < 0.001, Figure 4C), EBNA1 mRNA was detected by qRT-PCR. When EBNA1 was lacked, CYPA knockdown did not affect luciferase activity of oriP-SV40-Luc (Figure 4C). The results indicated that CYPA enhanced the EBNA1 activation of oriP transcription. The ChIP assay was used to further validate that CYPA played a role in EBNA1-oriP binding. pCAGGS-Myc-EBNA1 and oriP-SV40-Luc were co-transfected into HEK293-shNC and HEK293-shCYPA cells. As shown in Figure 4D, CYPA interference (shCYPA) reduced the EBNA1 binding to oriP DNA by approximately 50% compared to that of the control (shNC) in HEK293 cells (*P* < 0.05).

### The PPIase Activity of CYPA Is Required for Its Role in Viral Replication

**FIGURE 5:**
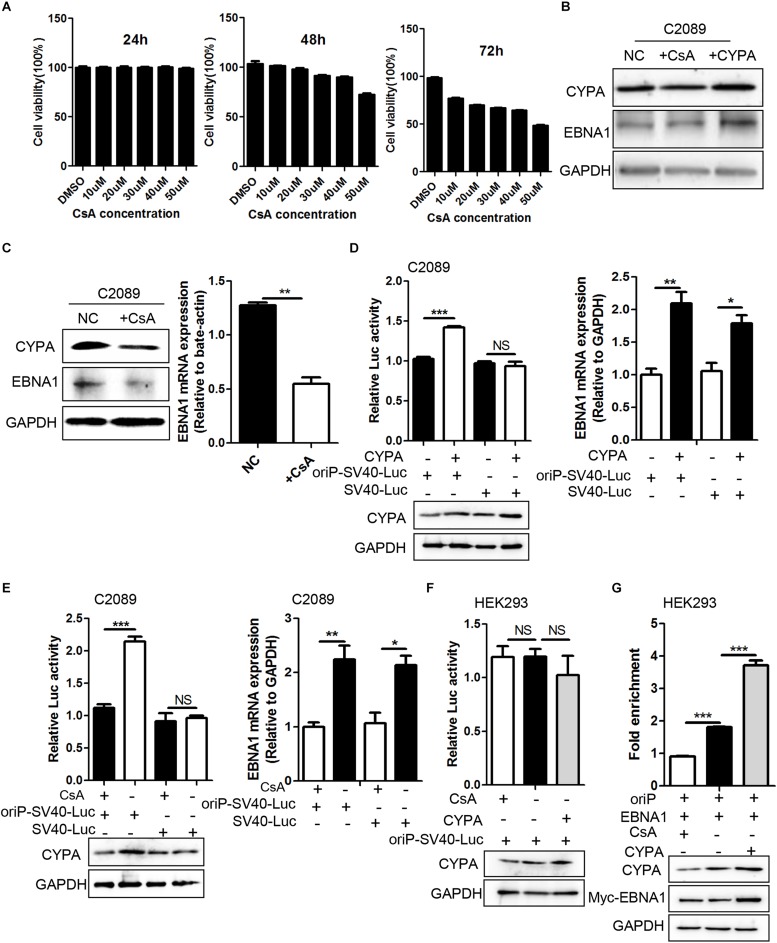
The effect of CYPA-specific inhibitor CsA on EBNA1–mediated oriP transcription and EBNA1-oriP binding. **(A)** The concentration determination of CsA by Cell Counting Kit-8 (CCK-8) assay. The concentration of 40 μM at 48 h was determined as the working concentration for treatment. **(B)** Rescue experiment of EBNA1 protein level after CsA treatment in C2089 cells. **(C)** The protein expression of CYPA and EBNA1 detected by WB assay at 48 h post-treatment with CsA. The mRNA expression of EBNA1 measured by RT-qPCR. EBV-positive C2089 cell lines were used for the test. **(D)** C2089 cells were transfected with oriP-SV40-Luc reporter plasmid and CYPA expression plasmids. Overexpressed CYPA significantly increased EBNA1- oriP-dependent luciferase activity, but had no effect on SV40 promoter dependent luciferase activity (left). EBNA1 mRNA was measured by RT-qPCR (right). **(E)** OriP-SV40-Luc reporter plasmid was transfected into C2089 cells, following the CsA treatment. CsA treatment greatly reduced EBNA1- oriP luciferase activity compared with untreatment. **(F)** CsA and elevated CYPA on EBNA1-oriP-mediated transcription activity in the luciferase reporter assay in HEK293 cells. **(G)** ChIP- qPCR was used to determine EBNA1-oriP binding. CsA treatment reduced EBNA1-oriP binding while elevated CYPA increased binding. CYPA and EBNA1 proteins were analyzed by WB. ^∗^*P* < 0.05, ^∗∗^*P* < 0.01, ^∗∗∗^*P* < 0.001.

CYPA is an intracellular receptor of CsA, which in turn inhibits the PPIase activity of CYPA through binding to the hydrophobic pocket of CYPA (Chatterji et al., 2009). Based on above data about CYPA regulation in EBV genome transcription initiation, we further investigated the possibility of CsA against EBV replication. The working concentration of CsA was considered as 40 μm according to its inhibition effectiveness and low cytotoxicity by the Cell Counting Kit-8 assay (Figure 5A). Rescue experiments showed that EBNA1 protein levels were restored compared to the CsA treatment group (Figure 5B). EBNA1 protein expression was evaluated by WB after treated or untreated with CsA (Figure 5C, left). RT-qPCR analysis showed that there was a significant reduction for the EBNA1 following the decrease of CYPA caused by CsA (Figure 5C, right). Subsequently, C2089 cells were transiently transfected with the CYPA expression plasmid together with oriP-SV40-Luc. Luciferase activities were increased at 24 h post-transfection compared with that of no CYPA overexpression, but there was no change for SV40-Luc (without oriP), and EBNA1-oriP-Luc activity increased in CYPA overexpression (Figure 5D). The protein level of CYPA and the mRNA level of EBNA1 are detected (Figure 5D).

We further investigate the role of CsA in EBNA1-mediated oriP transcription. After transfection of oriP-SV40-Luc, the cells were treated with CsA. The result showed that CsA decreased EBNA-oriP-Luc activity compared to the untreated group, with a decrease in CYPA and EBNA1 (Figure 5E). However, overexpression of CYPA and treatment of CsA in HEK293 cells did not affect the activity of oriP-SV40-Luc (Figure 5F). These data indicated that CsA inhibited the EBNA1-mediated oriP activation. ChIP assay followed by quantitative PCR (ChIP-qPCR) was used to evaluate the effect of CYPA overexpression and CsA treatment on EBNA1 binding to oriP in HEK293 cells. Myc antibody (for Myc-EBNA1) efficiently pulled down oriP-DNA from the above samples, and the results showed that CsA treatment eliminates EBNA1-oriP binding, while CYPA overexpression increases its binding (Figure 5G). The results suggested that the PPIase activity of CYPA was required for the role of CYPA in EBV latent replication.

### CYPA Overexpression Antagonizes EBNA1-USP7 Binding

**FIGURE 6:**
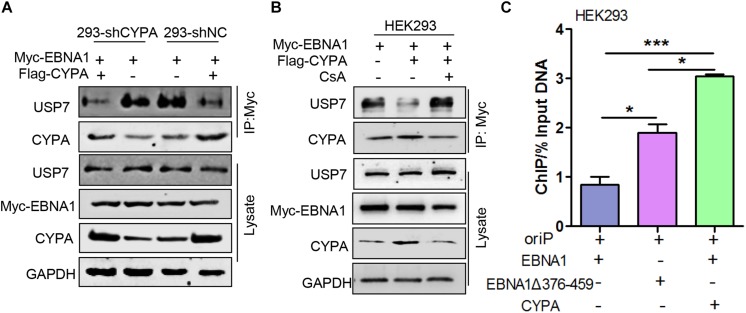
The effect of ectopic CYPA expression on USP7-EBNA1 and EBNA1-oriP binding. **(A)** Effect of CYPA overexpression on EBNA1-USP7 binding in HEK293-shCYPA and HEK293-shNC cells. These cells were transiently transfected with pCAGGS-Flag-CYPA alone or with pCAGGS-Myc-EBNA1, a co-IP assay was performed with the anti-Myc antibody for the pulldown. **(B)** The inhibition of CsA on CYPA-USP7 antagonism in the binding with EBNA1. pCAGGS-Myc-EBNA1 was transfected with pCAGGS-Flag-CYPA or alone, and treated with CsA. The anti-Myc antibody was used for the pulldown, and proteins were detected by WB. **(C)** Effect of CYPA overexpression and EBNA1Δ376-459 mutation on the EBNA1-oriP binding detected by ChIP assay. ^∗^*P* < 0.05, ^∗∗∗^*P* < 0.001.

Ubiquitin-specific protease 7 has been implicated in strong inhibition of EBV replication through its tight connection with EBNA1 (Holowaty et al., 2003b). In this study, as the above results demonstrated, the CYPA binding domain was located within the domain containing amino acids 376-459, which spanned the USP7-binding domain in EBNA1. Because CYPA played an opposite role in regulating EBNA1 function compared with USP7, HEK293-shNC and HEK293-shCYPA cells were transiently transfected with pCAGGS-Flag-CYPA alone or with pCAGGS-Myc-EBNA1, a co-IP assay was carried out. The results showed that USP7-EBNA1 binding was strong in the HEK293-shCYPA and HEK293-shNC cells. However, when CYPA was overexpressed, the amount of USP7 bound with EBNA1 was decreased remarkably (Figure 6A). In order to study the PPIase activity of CYPA in this mechanism in EBV replication, a co-IP assay was performed to using the treatment of CsA. The result verified that CsA eliminated the antagonism of CYPA on EBNA1-USP7 interaction (Figure 6B).

To investigate the ability of CYPA to influence the EBNA1-oriP connection through antagonizing USP7, in HEK293 cells, oriP-SV40-Luc was transfected with pCAGGS -Myc-EBNA1, pCAGGS-Flag-CYPA or pCAGGS-Myc-EBNA1 mutant, a ChIP assay was designed based on ectopic CYPA expression. As shown in Figure 6C, the interaction of oriP and the EBNA1 mutant with deletion of amino acids 376-459 (EBNA1Δ376-459) was significantly enhanced. In contrast, CYPA overexpression increased EBNA1-oriP binding to a high level. The result demonstrated that the deletion of the binding site for both USP7 and CYPA exhibited the enhancement of EBNA1-oriP binding (Figure 6C). This was a priority effect of the release of USP7 inhibition but not CYPA facilitation. Only when CYPA was overexpressed, could the effect of USP7 inhibition be reversed. The results further showed that CYPA overexpression was essential to overcome the USP7 suppression in regulating EBNA1 replication function.

## Discussion

**FIGURE 7:**
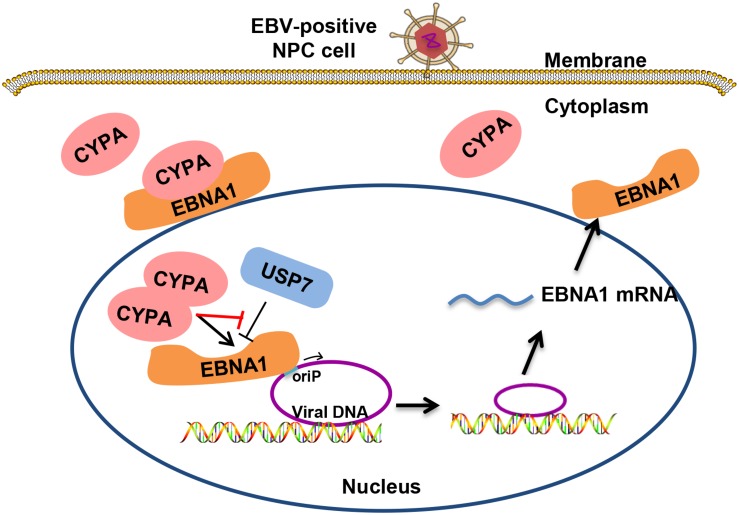
Schematic for the mechanism of CYPA in supporting the replication function of EBNA1. EBV genomic DNA exists within the host genome in the form of extrachromosomal episomes. Cytoplasmic CYPA can be hijacked by EBNA1 into the nucleus. Overexpressed CYPA can overcome the suppression of USP7 in binding to EBNA1. Nuclear CYPA mediates EBNA1-oriP transcription, and thus contributing to the viral genome replication and maintenance.

EBV latent infection is an important causative factor in the development of related cancers such as NPC (Dittmer et al., 2008; Zheng et al., 2014, 2018), although the mechanism is largely unclear. Viral replication and genome maintenance in host cells are important for the pathogenesis. CYPA was found to be highly expressed in NPC in the previous work from our laboratory (Yang et al., 2014; Liu et al., 2019) and played an unknown role related to EBV. Herein, we reveal that CYPA, especially when increasingly expressed, contributes to the replication function of EBNA1. CYPA first was recruited by EBNA1 into the nucleus and then mediated EBNA1-oriP binding and replication activity. On the other hand, when CYPA was upregulated, the USP7 suppression in EBNA1-mediated replication could be reversed. This interaction is a type of quantity-driven winning for CYPA, because its rival USP7 is too powerful. It was reported that the affinity of USP7-EBNA1 was 10-fold higher than USP7-p53, implying the strong binding of USP7-EBNA1 (Saridakis et al., 2005). The CsA treatment demonstrated that the PPIase activity of CYPA was required for this function. The schematic working model is shown as in Figure 7.

Cyclophilin A has been implicated in the life cycles of several viruses and plays a critical role in the successful infectivity and replication of these viruses, including some tumor viruses, such as HBV and HCV (Bose et al., 2003; Naoumov, 2014; Jyothi et al., 2015; Phillips et al., 2015). It has not been reported for the relationship between CYPA and KSHV, another tumor virus in the same family of gamma herpesvirus as EBV. Though CYPA is involved in the regulation of several viruses, its function mode is different from that in other viruses depending on the different mode of viral infection and replication. For example, the interaction between CYPA and HIV Gag was proposed to facilitate disassembly of the viral RNA containing core following virus entry and thus supports the efficient reverse transcription of the HIV-1 genome (Luban et al., 1993; Ott, 2002) CYPA also enhances virus attachment to the host cell membrane through interactions with heparans (Saphire et al., 2000) and after membrane fusion through interaction with CD147, thereby promoting viral infection. In the present study, for the first time, we showed that CYPA was also utilized by EBV in the modulation of viral replication function in epithelial cells. Thus, CYPA is involved in the maintenance of the virus during its latency in host cells. EBNA1 mediates DNA episome replication from oriP (Frappier, 2012a). Our results revealed that CYPA was recruited to influence EBNA1-oriP-mediated transcription. CYPA depletion significantly facilitated loss of EBV copy numbers (Figure 3F), suggesting that impairment of EBNA-oriP binding further weakened successful replication and maintenance of the EBV genome.

Epstein-Barr virus can naturally infected B cells and latently maintained in resting B cells (Thorley-Lawson et al., 2013). As CYPA is a multi-functional protein, which can be expressed in all kinds of cells, and EBNA1 is the only expressed protein of EBV in all latency types. Therefore, we think that both CYPA and EBNA1 may be expressed in resting B cells. How CYPA plays a role in the function of EBV in B cells remains to be further investigated.

The deubiquitinase USP7 is also known as a HAUSP and has been found to be associated with several herpesviruses, including HSV-1, EBV, and KSHV (Holowaty et al., 2003b; Jager et al., 2012; Hammerschmidt and Sugden, 2013). A previous study demonstrated that USP7 suppressed EBV replication (Holowaty et al., 2003b). Here, we showed that EBV hijacked the host factor of elevated CYPA to counteract USP7, thereby adding to the definition of HAUSP. Additionally, our data showed that deletion of the binding domain in EBNA1 for both USP7 and CYPA resulted in significantly increased EBNA1-oriP binding activity (Figure 6C), mainly due to release of USP7 inhibition. The result was consistent with that of previous report (Holowaty et al., 2003a), demonstrating that the suppressive role of USP7 was strong enough to greatly overshadow the improved role of CYPA.

In summary, the study reveals that CYPA is a novel critical host factor utilized by EBNA1 in the viral DNA replication in epithelial cells. Elevated CYPA levels remarkably antagonize USP7 in the interaction with EBNA1. The results revealed a strategy that EBV recruited a host factor to counteract the host defense, thus facilitating its own latent genome replication and efficient persistence. Our findings implied that EBV has evolved sophisticatedly. This study provides a new insight into EBV pathogenesis and potential virus-targeted therapeutics in EBV-associated NPC, in which CYPA is upregulated.

## Data Availability Statement

The data used to support the findings of this study are available from the corresponding author upon request.

## Author Contributions

SX, SD, and JL designed and conceived the experiments. JL supervised the research. SX, SD, LL, YX, LZ, JY, JH, WY, JZ, and PC performed the experiments and analyzed the data. SX, FZ, and JL wrote and revised the manuscript.

## Conflict of Interest

The authors declare that the research was conducted in the absence of any commercial or financial relationships that could be construed as a potential conflict of interest.

## Supplementary Material

FIGURE S1 The schematic diagram of the BiMC assay.

FIGURE S2 CYPA expression was detected by IF assay in Vero and HEK293T cells. Scale bar, 100 μm. After transfected the pCAGGS-Flag-CYPA plasmid for 24 h in Vero cells, the Flag antibody was incubated overnight in 4°C, followed by green fluorescent secondary antibody in 37°C for 1 h, hoechst33342 stained nucleus. HEK293T cells, CYPA antibody incubated overnight, followed by red fluorescent secondary antibody, stained nucleus.

FIGURE S3 Detection of EBNA1 mutants and CYPA using BiMC assays. **(A)** Detection of the interaction between each mutant and CYPA by BiMC assay. Scale bar, 50 μm. **(B)** Verification of the interaction between the single EBNA1 domain containing aa 376-459 and CYPA by the BiMC assay. Scale bars, 50 μm. **(C)** Negative controls for each single plasmid in BiMC assay.

TABLE S1 Primer sequences used in the study.
