# Supplementary figures and images for: Epstein-Barr Virus Nuclear Antigen 1 Recruits Cyclophilin A to Facilitate the Replication of Viral DNA Genome
Source: Front Microbiol. 2019 Dec 13;10:2879. doi: 10.3389/fmicb.2019.02879 (PMC6923202; doi:10.3389/fmicb.2019.02879)

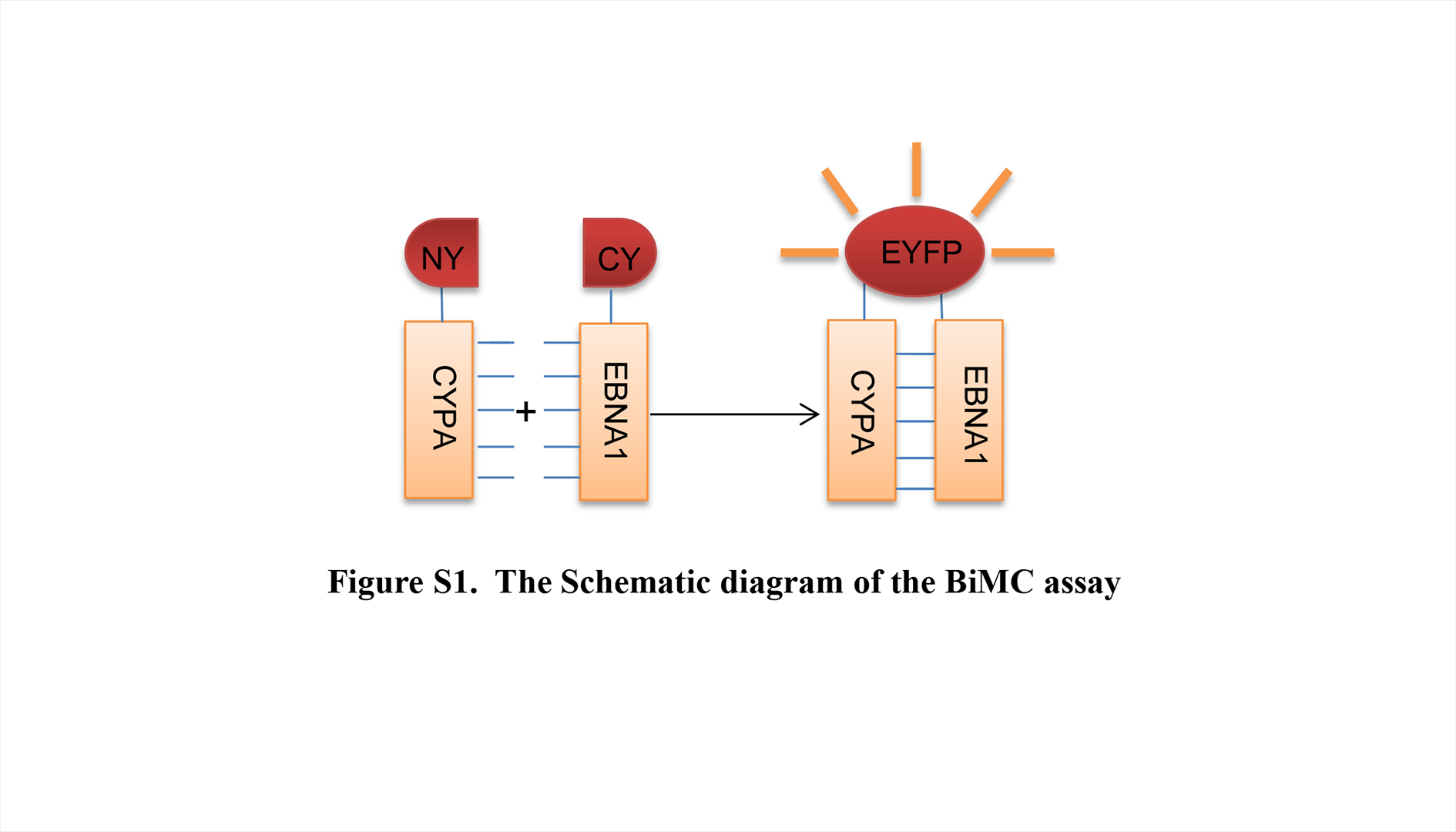

Supplement: FIGURE S1 — The schematic diagram of the BiMC assay. [file Image_1.TIF]

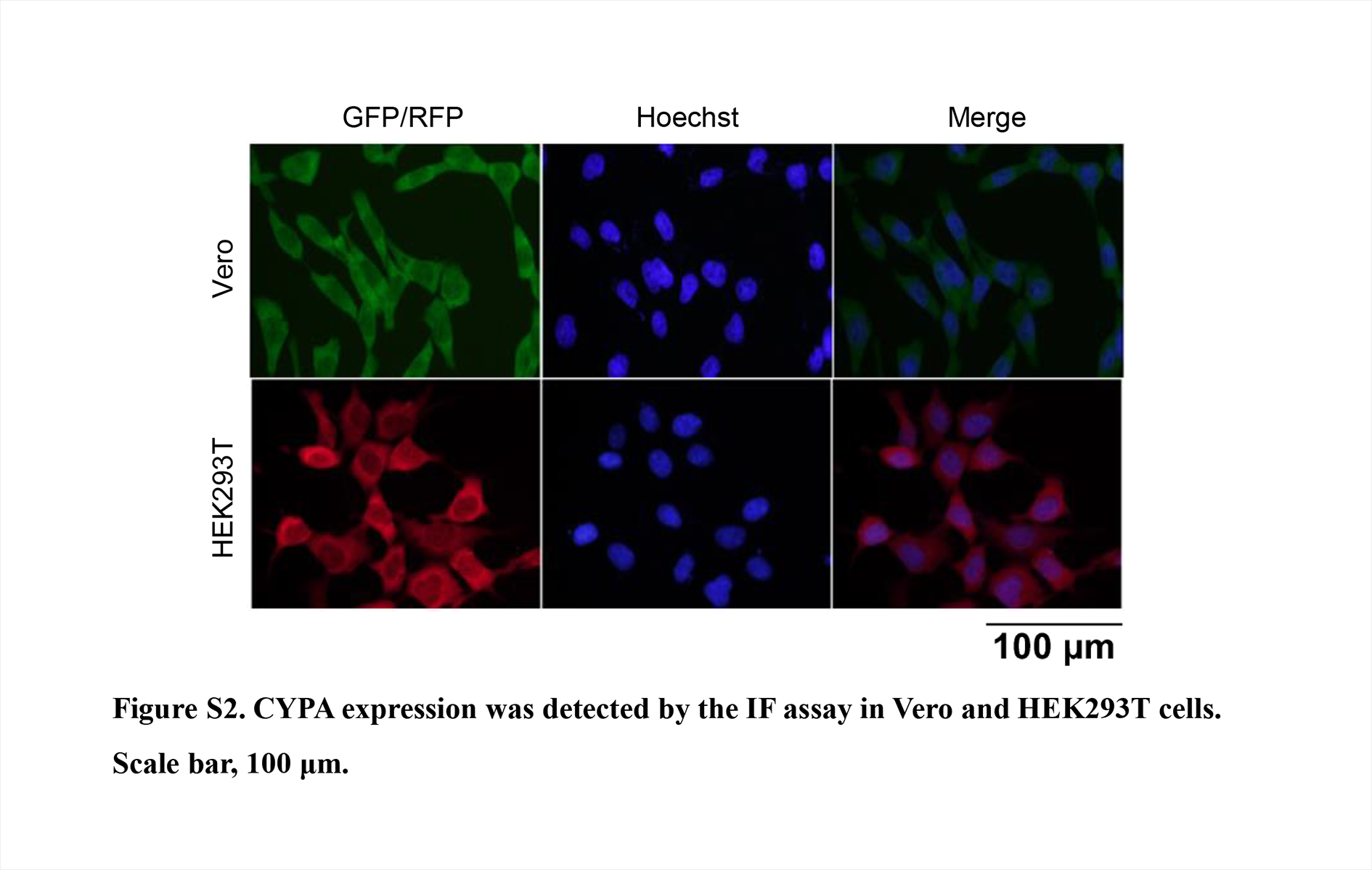

Supplement: FIGURE S2 — CYPA expression was detected by IF assay in Vero and HEK293T cells. Scale bar, 100 μm. After transfected the pCAGGS-Flag-CYPA plasmid for 24 h in Vero cells, the Flag antibody was incubated overnight in 4°C, followed by green fluorescent secondary antibody in 37°C for 1 h, hoechst33342 stained nucleus. HEK293T cells, CYPA antibody incubated overnight, followed by red fluorescent secondary antibody, stained nucleus. [file Image_2.TIF]

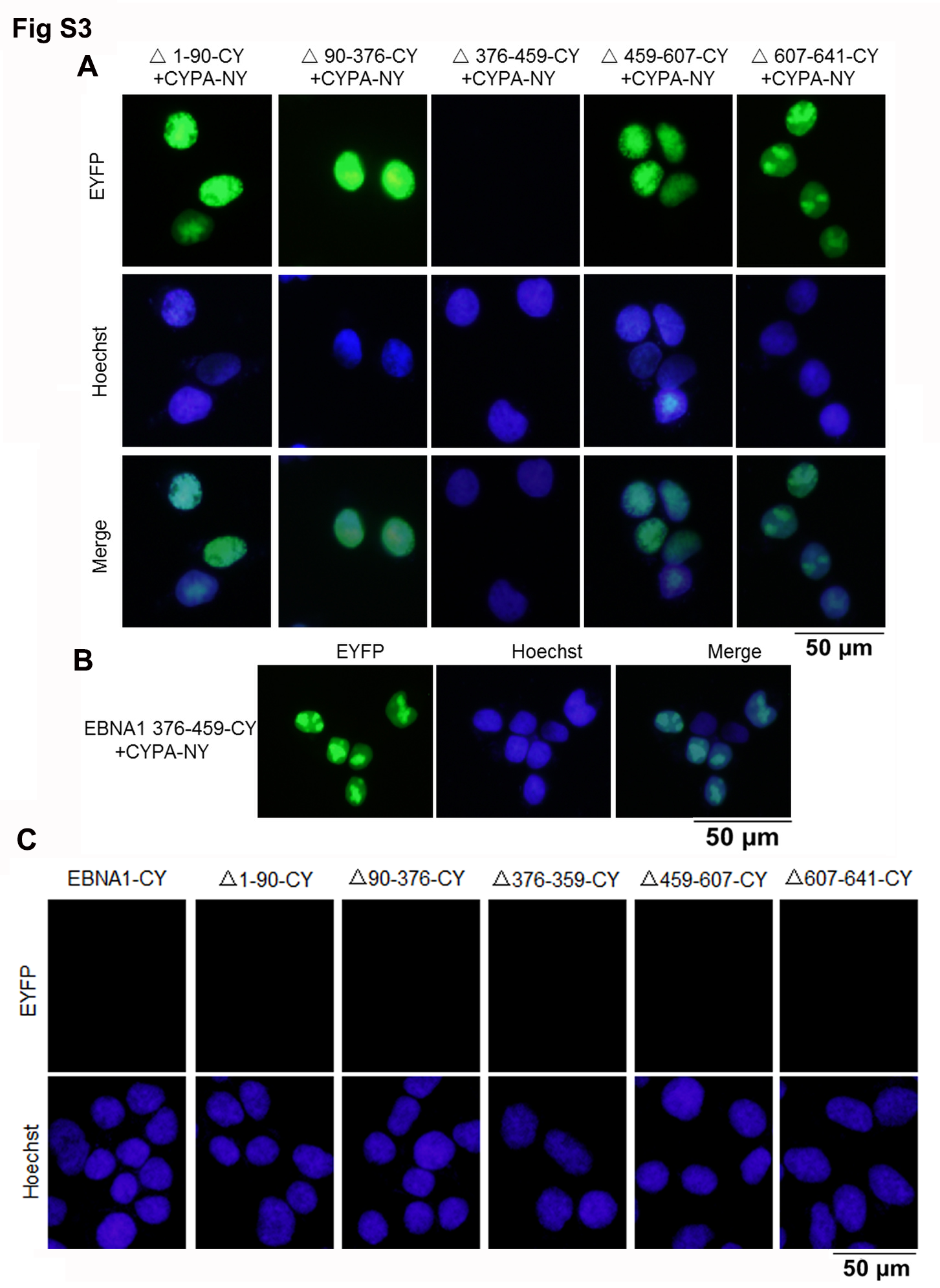

Supplement: FIGURE S3 — Detection of EBNA1 mutants and CYPA using BiMC assays. (A) Detection of the interaction between each mutant and CYPA by BiMC assay. Scale bar, 50 μm. (B) Verification of the interaction between the single EBNA1 domain containing aa 376-459 and CYPA by the BiMC assay. Scale bars, 50 μm. (C) Negative controls for each single plasmid in BiMC assay. [file Image_3.TIF]
