# Supplementary material for: Epstein-Barr Virus Nuclear Antigen 1 Recruits Cyclophilin A to Facilitate the Replication of Viral DNA Genome
Source: Front Microbiol. 2019 Dec 13;10:2879. doi: 10.3389/fmicb.2019.02879 (PMC6923202; doi:10.3389/fmicb.2019.02879)
Supplement: TABLE S1 — Primer sequences used in the study. [file Table_1.pdf]

Supplemental Table 1: Primer sequences used in the study

Primer pair No	Primer name	Primer sequences (5′-3′)
1	CYPA	FP GACTGAGTGGTTGGATGGCA<br>RP GCATGGGAGGGAACAAGGAA
2	CYPB	FP:AATA GAATTC ATGCTGCGCCTCTCCGAACG<br>RP:CGAT CTCGAGCTTATCGTCGTCATCCTTGTAATCCTCCTTGGCGATGGCAAAGG
3	oriP-SV40-Luc	FP CACGGCTAGCGAATTCTATCATTAAACGGC<br>RP GCCCAAGCTTCCTTTATGTGTAACTCTTGG
4	EBNA1△1-90	FP AATAGCATGCGGAGCAGGAGCAGGAGCGGG<br>RP ACTAGCTAGCTCACTCCTGCCCTTCCTCAC
5	EBNA1△90-376	FP GGAGAAAAGAGGCCCAGGAGTCCCAGTA<br>RP TGTTCCACCGTGGGTCCCTTTGCAG
6	EBNA1△376-459	FP CGCAAAAAAGGAGGGTGGTTTGGAAAGC<br>RP ACGTCCACGACCTCTCCCCCTGGCTCTT
7	EBNA1△459-607	FP CCTCCCTGGTTTCCACCTATGGTGGAAG<br>RP CCTGCCTCCATCACCCTGACCCCG
8	EBNA1△607-641	FP ACATGCATGCGAGCAAAAGCTCATTTC<br>RP CCTAGCTAGCAGGCAAATCTACTCCATCGTC
9	CYPA(qRT)	FP GACTGAGTGGTTGGATGGCA<br>RP GCATGGGAGGGAACAAGGAA
10	EBNA1(qRT)	FP CATCATCATCCGGGTCTCCA<br>RP ACGATGCTTTCCAAACCACC
11	LMP1(qRT)	FP TGAACACCACCACGATGACT<br>RP GTGCGCCTAGGTTTTGAGAG
12	bate-actin(qRT)	FP GCATCCCCCAAAGTTCACAA<br>RP AGGACTGGGCCATTCTCCTT
13	oriP(ChIP)	FP ATGTAAATAAAACCGTGACAGCTCAT<br>RP TTACCCAACGGGAAGCATATG
